# Comparative Stress Levels among Residents in Three Chinese Provincial Capitals, 2001 and 2008

**DOI:** 10.1371/journal.pone.0048971

**Published:** 2012-11-12

**Authors:** Tingzhong Yang, Dan Wu, Weifang Zhang, Randall R. Cottrell, Ian R. H. Rockett

**Affiliations:** 1 Center for Tobacco Control Research, Zhejiang University School of Medicine, Hangzhou, Zhejiang, China; 2 Health Promotion and Education Program, School of Human Services, University of Cincinnati, Cincinnati, Ohio, United States of America; 3 Injury Control Research Center and Department of Epidemiology, School of Public Health, West Virginia University, Morgantown, West Virginia, United States of America; Wayne State University, United States of America

## Abstract

**Objectives:**

To compare stress levels among residents in large Chinese cities between 2001 and 2008.

**Methods:**

Survey data were collected in three mainland Chinese capital cities in two waves, in 2001 and 2008, respectively. Participants were recruited through a multi-stage stratified sampling process. Stress was assessed using the Perceived Stress Scale, Chinese version (CPSS). Descriptive methods were used to estimate mean stress levels and associated 95% confidence intervals. Estimates were adjusted by post-stratification weights.

**Results:**

Indicating stable stress levels, respective adjusted mean stress scores for the combined samples of study participants were 23.90 (95%CI: 23.68–24.12) in 2001 and 23.69 (95%CI: 23.38–24.01) in 2008. A lower stress level in 2008 than in 2001 manifested among residents who were under 25 years of age; female; with a college or higher level education; divorced, widowed, or separated; members of the managerial and clerical group; students or army personnel; or with an annual income of at least 30,000 RMB.

**Conclusion:**

The overall stress level did not change among the combined sample of residents in the three Chinese study cities between 2001 and 2008. However, levels remained high and varied across social strata, and may have reflected a national trend among urban residents. Findings indicate a need for a new health policy, and call for the design and implementation of evidence-based interventions that target the highest-risk groups.

## Introduction

Since 1978, China has been transitioning from a centralized to a market-based economy. A concomitant is massive social change [1]–[3]. This socioeconomic transition has promised higher living standards, and markedly improved individual choice in education, health, consumer goods and services, and employment. It simultaneously has generated many challenges, including an imbalance between urban and rural development, rampant corruption, and a chasm between rich and poor [4], [5]. Studies suggest that people exposed to such conditions endure high stress [6]–[8]. For example, one study reported that 64% of urban residents manifested moderate or high levels of stress, and 22% suffered from mental disorders [7].

Stress is now a major public health problem in China, with an estimated 173 million Chinese adults having a mental disorder [6]. Many studies show escalation of stress-related health problems [6], [7]. The World Health Organization (WHO) estimated that neuropsychiatric conditions and suicide collectively comprised over 20% of the total illness burden of China in 2004 [9]. Given the huge population, even a conservative estimate gives China the largest number of reported suicides in the world [10]. Chinese suicides account for between 25% and 33% of the official global total, with at least 600–800 people committing suicide daily. In recent decades, there has been a striking increase in alcohol consumption and related problems in China [11], [12]. However, most Chinese stress studies have been based on a single cross-sectional survey that was confined to local subpopulations [13]–[15]. There is a crucial need to collect national population-based, longitudinal data on stress in urban communities with multiple time points, in order to inform health policy, plan prevention strategies, and design and implement evidence-based and targeted interventions. This research examined changes in stress levels in three Chinese provincial capital cities for two time points, 2001 and 2008, in addressing this information gap.

## Methods

### Study Design

This observational study used a cross-sectional multistage sampling design. A common survey instrument was administered in the same set of three large mainland Chinese cities in March, 2001, and May, 2008. In stage one, city selection was determined by geographical location. These cities were Taiyuan, Hangzhou, and Guangzhou, and all are capitals of their respective provinces. They represent three distinct geographic areas of China, with one being located in the north, another in the middle of the country, and the third in the south. Tiayuan (3.46 million people) is known for manufacturing, Hangzhou (6.72 million) is characterized by light industry and tourism, and Guangzhou (7.74 million) features light industry and commercial development [16].

Stage 2 comprised the selection of residential districts within each study city. Two residential districts, excluding new building districts and subdistricts, were randomly selected from the sampling frame of residential districts within each city. In Stage 3, four ‘Jiedao’ (a subdistrict neighborhood) were randomly selected from each residential district, and 16 building blocks were randomly selected from each ‘Jiedao’. In Stage 4, a family household registration (“hukou”) list was used to randomly sample households within the selected building blocks. Individuals aged 15 years and older, who had lived in their home for at least one year, were identified within each household. Fifteen was selected as our age cutoff to be consistent with previous research on stress and behavioral issues in China (23). However, age 18 years marks the official onset of adulthood in China. In the fifth and final stage, one household member whose birthday was closest to the contact date was selected for interview.

### Interview and Human Subjects Procedures

A face-to-face interview was scheduled once an individual was identified and agreed to participate. All interviews were conducted privately by trained interviewers. A common interview protocol was utilized across the three study cities and two survey time points, in order to achieve homogeneity in interview and data collection procedures. The study was approved by the Ethics Committee at the Zhejiang University Medical Center, and informed written consent was obtained from all participants prior to their interview.

### Measures

A structured questionnaire was used in collecting data on stress levels and the following socioeconomic characteristics: age, gender, marital status, educational level, occupational or labor force status category, and income. Stress was measured on the Perceived Stress Scale, Chinese version (CPSS) [7], [17], [18]. This scale comprised 14 items that assessed perception of stress during the month prior to survey. Items were rated on a 5-point Likert-type scale, and ranged from 0 (never) to 4 (very often). Item scores were summed to yield a total stress score. The higher the total score, the greater the perceived level of stress [7], [17], [18]. Following prior practice, severe stress was operationalised as a score > = 25 [7].

### Data Analysis

Differences in mean stress scores for 2001 and 2008 were calculated using SPSS version 17. We first weighted our sample by city population [16]. We did not consider district, subdistrict, blocks of apartment buildings, or households in our weighting scheme, since differences in these characteristics were minimal within a given city [19]. Secondly, we implemented a post-stratification weight adjustment for participant gender and age in each of the three study cities [20]–[22], based on estimated distributions of these characteristics from a national post-enumeration survey [23]. These variables were simultaneously adjusted for in the statistical analysis. The final weight assigned to each participant was the product of the sampling and stratification weights [21], [22]. We conducted a post-stratified multistage survey sampling-based analysis using the SPSS Complex Samples procedure.

## Results

For the 2001 survey, 3246 individuals were contacted, with 3034 (93.5%) completing the interview: 1234 (40.6%) from Hangzhou, 932 (30.7%) from Guangzhou, 868 (28.6%) from Taiyuan. In the 2008 survey, 2435 individuals were contacted, and 2250 (92.4%) completed the interview: 797 (35.4%) from Hangzhou, 695 (30.9%) from Guangzhou, and 758 (33.7%) from Taiyuan. To be considered a participant, all questions on the survey had to be answered. Non-participants either provided no information of any kind or incomplete information. Table 1 shows some demographic variation in the urban samples across the two survey waves. The unadjusted mean stress scores were 24.14(95%CI: 23.93–24.34) in 2001 and 22.67(95%CI: 22.49–22.91) in 2008 indicated a decline in the overall stress level. With adjustment for demographic variation, stress levels between 2001 (23.90; 95%CI: 23.68–24.12) and 2008 (23.69; 95%CI: 23.38–24.01) were stable.

**Table 1:** Demographic characteristics of survey samples, 2001 and 2008.

	2001 (N: 3034)		2008 (N: 2250)		X^2^	P
	N	weighted %	N	weighted %		
***Age*** (in years)					624.016	0.000
<25	291	9.2	532	23.6		
25–34	786	25.9	578	25.7		
35–44	975	32.1	327	14.5		
45–54	695	22.9	390	17.3		
55+	287	9.5	423	18.9		
***Gender***					5.860	0.016
Male	1642	54.2	1293	57.5		
Female	1392	45.8	957	42.5		
***Education***					80.751	0.000
Junior high school or lower	775	25.6	727	32.3		
High school	1307	43.1	703	31.2		
Junior college	561	18.5	453	20.1		
College or higher	391	12.9	367	16.3		
***Marital status***					402.983	0.000
Never married	376	12.4	774	34.4		
Married	2589	85.3	1380	61.3		
Other (divorced, widowed, separated)	69	2.3	96	4.3		
***Occupation/Labor force status***					464.623	0.000
Managers and clerks	929	30.6	188	8.3		
Professionals	296	9.8	222	9.9		
Commercial and service workers	534	17.6	358	15.9		
Operational workers	497	16.4	468	20.8		
Students or army personnel	127	4.2	160	7.1		
Unemployed	102	3.3	121	5.4		
Retirees	279	9.2	350	15.6		
Others	270	8.9	383	17.0		

When considering the stress scores for our urban sample, adjusted for region, age, and gender across the two waves, they were respectively lower in 2008 than 2001 for residents who were under 25 years of age; female; with a college or higher level education; divorced, widowed, or separated; members of the managerial and clerical group; students or army personnel; or with an annual income of at least 30,000 RMB. In 2001, residents under age 25 showed a higher stress level than all older groups, but there was no age variation in 2008 (Table 2). Males were less stressed than females in 2001, but no gender variation manifested in 2008. In 2001, urban residents with at least a college education were less stressed than the least-educated group, namely, those with a junior high school or less education. In 2008, their stress level was lower than all of the lesser-educated groups. The divorced, widowed and separated group was less stressed than the married and never married in 2001. In 2008, stress levels did not vary by marital status. Turning to occupational and labor force status, in 2001 professionals, managers and clerks, commercial and service workers, and operational workers had lower stress levels than students or army personnel, and retirees had a higher level than professionals, managers and clerks. In 2008, managers and clerks showed less stressed than all comparative groups, except professionals and the student/army group. In 2001, urban residents earning 40,000 RMB annually were less stressed than those whose income was under 20,000. In 2008, residents earning 30,000 RMB or more were less stressed than lower-income earners.

**Table 2:** Mean stress scores of combined residents from three large capital cities by demographic characteristics, 2001 and 2008#.

	2001 (N: 3034)			2008 (N: 2250)		
	n	Mean	95% CI	n	Mean	95% CI
***Age*** ** ***(in years)***						
<25	291	25.42	24.80–26.04	532	23.78	23.19–24.36*
25–34	786	24.32	23.91–24.73	578	23.63	23.04–24.21
35–44	975	24.00	23.64–24.37	327	24.15	22.33–24.96
45–54	695	23.56	23.11–24.00	390	23.65	22.88–24.43
55+	287	23.89	23.06–24.73	423	23.00	22.28–23.73
***Gender***						
Male	1647	23.49	23.21–23.78	1293	23.94	23.54–24.34
Female	1398	24.48	24.17–24.79	957	23.37	22.86–23.87*
***Education***						
Junior high school or lower	779	24.28	23.85–24.70	727	24.09	23.56–24.62
High school	1311	23.87	22.55–24.19	703	24.08	23.52–24.64
Junior college	563	23.47	23.01–23.94	453	23.04	22.32–24.75
College or higher	392	23.22	22.62–23.82	367	20.39	19.52–21.23**
***Marital status***						
Never married	378	24.67	23.52–24.62	774	23.68	23.16–24.20
Married	2598	23.90	23.67–24.13	1380	23.69	23.29–24.09
Other (divorced, widowed, separated)	69	26.50	25.19–27.81	96	23.33	21.84–24.82 *
***Occupation/Labor force status***						
Managers and clerks	932	23.51	23.14–23.89	188	21.38	20.16–22.61**
Professionals	297	23.46	22.85–24.06	222	22.93	21.91–23.95
Commercial and service workers	536	23.97	23.48–24.46	358	24.17	23.37–24.98
Operational workers	503	24.03	23.51–24.54	468	24.37	23.65–25.08
Students or army personnel	127	26.70	25.32–28.08	160	23.41	22.27–24.54**
Unemployed	101	24.08	22.72–25.44	121	25.07	23.70–26.44
Retirees	279	24.86	24.14–25.58	350	23.66	23.86–24.46
Others	270	23.52	22.66–24.38	383	23.70	23.00–24.39
***Income/person/year(RMB)***						
<10,000	634	24.05	24.18–25.10	650	24.12	23.54–24.71
10,000–19,999	735	24.05	23.59–24.58	733	24.90	24.42–25.38
20,000–29,999	617	23.80	23.37–24.22	436	24.03	23.36–24.72
30,000–30,999	505	23.88	23.36–24.40	181	21.79	20.60–22.98*
40,000+	554	22.91	22.41–23.41	250	19.87	18.76–20.95**

# Results were adjusted for region, age and gender.

* = p≤0.05.

** = P≤0.01.

## Discussion

This study examined differences in stress levels and related socioeconomic characteristics among residents comprising the combined urban samples from three large Chinese capital cities for years 2001 and 2008. Sample characteristics varied to some extent between the two survey waves, and this variation may reflect both these from sampling error, and true social change during this eight-year study period. The proportion of managers and clerks in the 2001 sample was higher than in the 2008 sample, a change that may indeed have been real. In the past, there was an overabundance of managers and clerks in government, schools, universities, and other social organizations. More recently, the Central Government initiated reforms aimed at increasing economic efficiency through eliminating surplus positions.

Our study found no statistically significant difference in the adjusted mean stress scores for urban residents in 2001 and 2008. Nevertheless, the stress level was relatively high in both years. The adjusted mean stress score in 2008 was 23.69, and 44.6% (95%CI: 42.5–46.7) of the residents showed severe stress. Stress levels appear very high among those in the lower socioeconomic groups in particular, and impediments to changing the stressful life situations of these people are manifold and formidable.

China has been rapidly transitioning from a centrally-planned to a market-oriented economy. In so doing, it initiated economic reform in rural areas that subsequently encompassed urban areas. Reform evolved through four stages. The first stage was the preparation period (1978–1987), the second stage the moderate period (1988–1994), and the third the radical period (1995–1999). The fourth and current stage, commencing in 2000, is known as the regulation period. Many social issues, which had manifested in the moderate period, intensified in the radical period. The radical period saw abolition or deregulation of numerous state-owned enterprises and heavy losses of menial jobs [4], [5], [15]. During the radical period, serious inequalities emerged. A minority of individuals controlled most of the resources. Disproportional sharing of financial resources induced vast income inequality among the various economic sectors and regions of China. This profound change from a period when resources were more equally distributed may have contributed to high stress levels among urban residents [5]. At the same time, modern lifestyles, with their strong social and time demands, may also have elevated stress levels [7]. In the current or regulation period, the Central Government has focused on building a more “Harmonious society,” with particular emphasis on protecting disadvantaged groups. Our study fell within this period, so we might have anticipated finding lower stress levels. That levels are persistently high is probably a product of accumulated stress that carried over from the radical period, or an indication that the government policies to create a “Harmonious Society” have so far been unsuccessful.

Social changes, including increased income inequality, continue to fuel high stress, a patently serious public health problem among Chinese urban residents [1], [4]–[7], [24], [25]. Prevention needs to target those urban residents who are at risk of severe stress due to low socioeconomic status. The Chinese government must acknowledge their special vulnerability, and create policies that both mitigate income inequality and guarantee economic benefits to the disadvantaged.

This study found lower stress levels in 2008 than in 2001 for urban residents under 25 years of age, females, those with higher educational attainment, and the divorced, widowed and separated. These findings may emanate from reforms and their differential socioeconomic impact. The significantly lower stress levels in 2008 than in 2001 for urban residents under 25 years of age may reflect better employment opportunities for younger people in the new market-oriented economy. Furthermore, there were large increases in university enrollment during this time [26], which has given more young people the opportunity to obtain advanced degrees and the concomitant benefits of higher education.

In 2001, females were more stressed than males. However, there was no difference in stress levels between females and males in 2008. This may indicate more gender equality in the wake of reform and development. A lower stress level was noted in 2008 than 2001 among the divorced, widowed, and separated, a finding suggesting greater social tolerance of marital disruption and dissolution, and sympathy for spousal loss [27]. The estimated prevalence of urban residents who were divorced, widowed, or separated grew from 2.3% in 2001 to 4.3% in 2008. The divorced, widowed, and separated were more stressed than the married and never married in 2001. However, in 2008 there was no stress variation by marital status. This finding may indicate greater acceptance and tolerance of marital disruption, marital dissolution, and widowhood.

Confirming prior findings, stress levels appeared variable with socioeconomic status [18], [28]. One indicator of such status is education. More highly educated residents were less stressed in 2008 than 2001, while there was no difference in stress levels among the lesser educated. This finding may emanate from reforms, increasing educational attainment and its capacity to attract superior job opportunities, more extensive social networks, greater personal freedom, and healthier and safer working environments [13], [17], [28]–[30]. In addition, the more educated urban residents may have acquired stronger coping mechanisms than the lesser educated for confronting both life stressors and the socioeconomic metamorphosis in China [7], [30]. A second socioeconomic indicator is income. Our study confirmed that urban residents with higher incomes experienced less stress than lower-income counterparts [30]–[34]. Higher incomes for people assure satisfaction of basic living needs and improve educational access, which in turn mitigate daily stressors and pressures induced by social change [30], [34], [35].

Another research question implicating socioeconomic status was whether stress levels rose or fell during the observation period within our amalgamated occupational and labor force status category. Managers and clerks, and students or army personnel were less stressed in 2008 than 2001. We suspect that managers and clerks acquired relatively greater socioeconomic benefits from the reforms than other groups, except professionals, which then diminished their stress [36]. Stress reduction among the student/army group likely reflects the large increases in university enrollment and related educational opportunities that have occurred in China since 1999 [26].

The adjusted mean perceived stress levels for 2001 and 2008, based on current status at each survey wave for the urban samples, suggest stable stress levels over the period [18], [24], [33]. Furthermore, levels approach the high-stress cutoff value of 25, and suggest a serious and persistent chronic stress status among residents of large Chinese cities. This situation likely harbors important implications for underlying physical disease and psychiatric disorders, and reveals a need for officials to consider disease prevention and health promotion as part of stress-reduction initiatives.

The results of our study strongly suggest the importance of combating persistent and relatively high stress levels among residents of large Chinese cities. The Central Government and local health authorities need to collaborate on policies for stress reduction and prevention of mental disorders. Income inequality must be reduced, and benefits made available to the large disadvantaged element of society. If socioeconomic gaps can be narrowed, stress and mental health problems will decline.

At the community level, a multifaceted approach to prevention must be integrated into healthcare programs. For example, local health authorities should offer educational and counseling programs as well as mental healthcare in hospitals and clinics. Community-based clinics should be established to provide high-risk groups with necessary psychological counseling. Establishing 24-hour telephone hotlines to enable people in crisis to talk with mental health professionals on an as needed basis could be helpful. Support groups should be established in urban community centers to provide a forum where residents can express mental health concerns and learn stress-management skills. Employees could be reached through worksite programs that are designed to educate them about stress-related symptoms, and provide them with the skills of personal stress management. Moreover, worksites should offer counseling to employees who are experiencing high stress levels and mental issues or, at a minimum, refer them to appropriate community agencies. Within schools and universities, students should be provided with a health education curriculum that informs them of the risk factors for severe stress, and equips them with coping strategies. A nationwide media campaign should be planned and implemented to educate the populace about the adverse health effects of stress. This campaign would optimally involve multiple media outlets, including television, radio, newspapers, and billboards.

This study has several limitations. First, the study population was drawn from three large provincial capital cities, and these cities may not represent all Chinese cities. However, to achieve a high degree of large-city representation, we were careful in our selection to utilize criteria adopted in previous studies, and to select cities from different geographic regions with variable economic development. Our survey was large-scale and study results are likely a strong indicator of the extent and degree of stress-related issues within populations of large Chinese cities. Secondly, although a common interview protocol was utilized across the three study cities and two survey time points, in order to achieve homogeneity in interview and data collection procedures, we cannot validate the attainment of complete homogeneity. Since interviewers were not observed during data collection, some variation in the administration of the protocol may have occurred. Thirdly, we evaluated stress by means of self-report, which may have induced a related bias. Fourthly, we only measured stress levels at two points in time, thus precluding an assessment of trends. For purposes of surveillance, prevention, and evaluation, it is essential to collect longitudinal data on stress in urban communities involving more frequent and multiple time-points. Additionally, community health promotion and policy data are required for comprehension of stress responses in the face of community-level health regulations and targeted interventions.

### Conclusion

This research contributes to the mental health literature by documenting stress levels among urban residents in China. In comparing urban residents in 2001 and 2008, we found an unchanging but high prevalence of stress. This finding underscores the importance for the Central Government and local health agencies to collaborate in developing and implementing a mix of policies, strategies, and programs aimed at stress reduction among urban Chinese residents. Countermeasures must be evidence-based, and embrace primary, secondary, and tertiary prevention. Stress is a common element in mental illnesses, as well as many major chronic health conditions [23], [28], [34], but there is a dearth of empirical research on this subject in China.
